# Therapie-Apps und digitale Gesundheitsanwendungen in der Behandlung der adulten Aufmerksamkeitsdefizit‑/Hyperaktivitätsstörung: ein Überblick

**DOI:** 10.1007/s00115-025-01855-1

**Published:** 2025-08-04

**Authors:** Marie-Christin Atzor, Luisa Jung, Benjamin Selaskowski, Max Pensel, Silke Lux, Alexandra Philipsen, Niclas Braun, Matthias Guth

**Affiliations:** Poliklinik für Psychiatrie und Psychotherapie, Universitätsklinikum Bonn, Venusberg-Campus 1, 53127 Bonn, Deutschland

**Keywords:** Mobile Interventionen (mhealth), Digitale Interventionen, Evidenz, Kernfunktionen, Mobile-Sensing-Funktionen, Mobile health (mhealth), Digital interventions, Evidence, Key functions, Mobile sensing functions

## Abstract

**Hintergrund:**

Digitale Interventionen, die flexibel im Alltag eingesetzt werden können, stellen potenziell eine wertvolle Ergänzung zu etablierten Therapieangeboten der adulten Aufmerksamkeitsdefizit‑/Hyperaktivitätsstörung (ADHS) dar.

**Fragestellung:**

In dieser Literaturübersicht werden die derzeit in Deutschland verfügbaren und sich aktuell in der Entwicklung befindlichen digitalen Interventionen für Erwachsene mit ADHS vorgestellt.

**Material und Methode:**

Ziel ist die Identifikation und Charakterisierung der für die adulte ADHS relevanten digitalen Interventionen mit Fokussierung auf deren Kernfunktionen, Nutzungsmodi sowie bisher erreichte Evidenz- und Reifegrade.

**Ergebnisse:**

Insgesamt wurden sechs Anwendungen für Erwachsene mit ADHS (hiFoon, attexis, ORIKO, AwareMe-App, AwareMe-Chatbot, NeuroNation MED) identifiziert. Für einen Teil der Interventionen wurden bereits klinische Studien mit ersten Wirksamkeitsnachweisen durchgeführt. Für andere sind klinische Studien geplant oder werden derzeit durchgeführt.

**Schlussfolgerungen:**

Eine der vorgestellten digitalen Interventionen lässt sich bereits als digitale Gesundheitsanwendungen (DiGAs) bei ADHS verordnen. Andere streben ebenfalls eine zeitnahe Listung im DiGA-Verzeichnis an. Innovationspotenziale bieten sich insbesondere in der Übertragung bestehender Anwendungen in neue Sprachen sowie in der Integration von Mobile-Sensing-Funktionen.

**Zusatzmaterial online:**

Die Online-Version dieses Beitrags (10.1007/s00115-025-01855-1) enthält eine Übersicht über die Begriffsdefinitionen sowie eine Tabelle zu digitalen Interventionen für Erwachsene mit Aufmerksamkeitsdefizit‑/Hyperaktivitätsstörung.

## Abstract (en)

**Background:**

Digital interventions for day-to-day use in adult attention deficit hyperactivity disorder (ADHD) can be a useful further treatment option in addition to established ADHD treatments.

**Objective:**

This review of the literature presents the digital interventions for adults with ADHD in Germany that are currently available or in advanced development.

**Material and methods:**

The aim is the identification and characterization of the digital interventions available for adult ADHD with a special focus on key features, modes of use, evidence achieved so far and maturity levels.

**Results:**

In total, six applications for adults with ADHD (attexis, AwareMe App, AwareMe Chatbot, hiFoon, NeuroNation MED, ORIKO) were identified. Clinical studies with initial proof of efficacy have already been carried out for some of the interventions, for the remainder clinical studies are planned or are currently being carried out.

**Conclusion:**

One of the digital interventions is already available on prescription in Germany as so-called digital healthcare apps (*digitale Gesundheitsanwendungen*, DiGA). Other developers plan to seek regulatory approval in the near future. Innovation potential is seen particularly in the translation of existing applications into new languages and in the future integration of mobile sensing functions.

Mit geschätzten Prävalenzraten von mehr als 2 % bei Erwachsenen hat die Aufmerksamkeitsdefizit‑/Hyperaktivitätsstörung (ADHS) eine hohe Relevanz für die Erwachsenenpsychiatrie. Im Rahmen einer leitliniengerechten Behandlung kommt neben der Psychoedukation und Stimulanziengabe auch der psychotherapeutischen Behandlung eine hohe Bedeutung zu. Ein wichtiger Fokus liegt dabei auf der Vermittlung von Bewältigungsstrategien zur Verbesserung der Funktionalität im Alltag. Digitale Interventionen, die flexibel im Alltag nutzbar sind, können in diesem Zusammenhang eine wertvolle Ergänzung zu etablierten Therapieangeboten bieten. Dieser Artikel gibt einen Überblick über derzeit verfügbare und sich aktuell in der Entwicklung befindliche digitale Interventionen für Erwachsene mit ADHS.

## Einführung

Digitale Gesundheitsanwendungen (DiGAs) im Bereich der Psychiatrie und Psychotherapie basieren überwiegend auf kognitiver Verhaltenstherapie und werden als störungsspezifische Onlinekurse entweder selbstständig oder therapiebegleitend genutzt. Zum Zeitpunkt dieser Übersichtsarbeit ist lediglich eine der vorgestellten Anwendungen als DiGA für ADHS verfügbar. Diese Übersichtsarbeit präsentiert deutschsprachige digitale Interventionen, die bereits eine Zulassung im DiGA-Verzeichnis (https://diga.bfarm.de/) erhalten haben oder diese anstreben. DiGAs sind CE-gekennzeichnete, vom Bundesinstitut für Arzneimittel und Medizinprodukte (BfArM) zugelassene Medizinprodukte, die von Ärzt:innen und Psychotherapeut:innen verordnet und von den Krankenkassen erstattet werden können. Es wird zwischen einer vorläufigen (max. 24 Monate) und einer dauerhaften Listung unterschieden. Während bei der vorläufigen Aufnahme im DiGA-Verzeichnis zunächst die Aussicht auf einen positiven Versorgungseffekt genügt, muss für ein dauerhafte Listung im DiGA-Verzeichnis ein Wirksamkeitsnachweis durch randomisierte kontrollierte Studien erbracht werden.

### ADHS – die wichtigsten Aspekte auf einen Blick

#### Symptomatik

Die Hauptsymptome der ADHS umfassen eine ausgeprägte Unaufmerksamkeit, Hyperaktivität und Impulsivität. Aktuelle Forschungsergebnisse zeigen, dass Hyperaktivität im Erwachsenenalter weniger häufig vorkommt. Stattdessen überwiegen Symptome wie Unaufmerksamkeit, innere Unruhe, Impulsivität und emotionale Dysregulation [11, 17].

#### Prävalenz

Im Erwachsenenalter liegt die Prävalenz der ADHS bei etwa 2,8 % [7], bei Kindern und Jugendlichen zwischen 4 und 5 % [19].

#### Bisherige Behandlung

Die Behandlung der ADHS erfolgt multimodal [4]. Die medikamentöse Therapie wird in der Regel mit Stimulanzien wie Methylphenidat oder Lisdexamfetamin eingeleitet, sofern keine bedeutsamen Kontraindikationen vorliegen. Eine mögliche Alternative ist Atomoxetin, ein noradrenerger Wirkstoff. Alle drei Substanzen sind in Deutschland für die Behandlung von Erwachsenen mit ADHS zugelassen [4]. In der ADHS-Therapie ist zudem eine umfassende Psychoedukation unabdingbar [4], welche über die Störung, ihre Ätiologie, Symptome und psychosoziale Auswirkungen sowie über geeignete Therapieoptionen und Copingstrategien aufklärt. Die Schwerpunkte einer Psychotherapie bei der ADHS liegen auf der Stärkung der Störungsakzeptanz, auf der Entwicklung funktionaler Bewältigungsstrategien, auf der Verbesserung alltäglicher Funktionen sowie auf der Resilienzförderung [4].

### Warum sind digitale Interventionen bei ADHS interessant?

Digitale Interventionen könnten sich als wertvolle Ergänzung in der ADHS-Behandlung etablieren [1, 6]: Sie sind örtlich und zeitlich flexibel nutzbar und können kostengünstig skaliert eingesetzt werden. Zudem können sie in verschiedenen Sprachen bereitgestellt werden. Auch ermöglichen sie einen breiten Zugang zu evidenzbasierter psychotherapeutischer Behandlung, auch ohne Wunsch nach klassischer Psychotherapie. Ebenso können digitale Interventionen psychotherapeutische Lerninhalte kleinteilig im Lebensalltag zur Verfügung stellen und dabei unterstützen, neue funktionale Verhaltensweisen in der täglichen Routine zu verankern und Symptome sowie Problemsituationen im Alltag aufzuzeichnen.

Digitale Interventionen lassen sich im Sinne eines Blended-care-Ansatzes gewinnbringend mit herkömmlichen psychotherapeutischen Angeboten kombinieren. Dies erscheint insbesondere für die Therapie der ADHS von Vorteil, bei der die praktische Umsetzung neuer Verhaltensstrategien, z. B. hinsichtlich der Aufmerksamkeitslenkung und des Problemlöseverhaltens, eine häufige Herausforderung darstellt.

## Aktuell verfügbare und sich in der Entwicklung befindliche Interventionen

Mit der Zunahme digitaler Gesundheitsinterventionen wächst auch das Angebot an digitalen Interventionen für Erwachsene mit ADHS. Im Folgenden werden verfügbare sowie in Entwicklung befindliche Interventionen hinsichtlich ihres Reife- und Evidenzgrads verglichen (Übersicht in Tab. e1 im Onlinezusatzmaterial).

### attexis

Die App attexis integriert therapeutische Techniken und Übungen zur Unterstützung des ADHS-Managements (https://www.attexis.de) und basiert auf einem kognitiv-behavioralen Ansatz. Sie bietet eine strukturierte, interaktive Behandlung in Form eines virtuellen Dialogs, bei dem die Nutzenden durch Auswahl vordefinierter Antworten auf Fragen des Programms den Interventionsverlauf beeinflussen.

#### Kernfunktionen und Nutzungsmodus

Die App umfasst zentrale Funktionen wie Psychoedukation, verhaltenstherapeutische Übungen zur Impulssteuerung und Organisation, Zielsetzung und Fortschrittsüberwachung sowie kognitive Strategien zur Verbesserung exekutiver Funktionen. Zudem bietet sie Verhaltenstracking zur Verfolgung täglicher Aktivitäten sowie Achtsamkeits- und Entspannungsübungen.

#### Evidenz- und Reifegrad

**Abb. 1:**
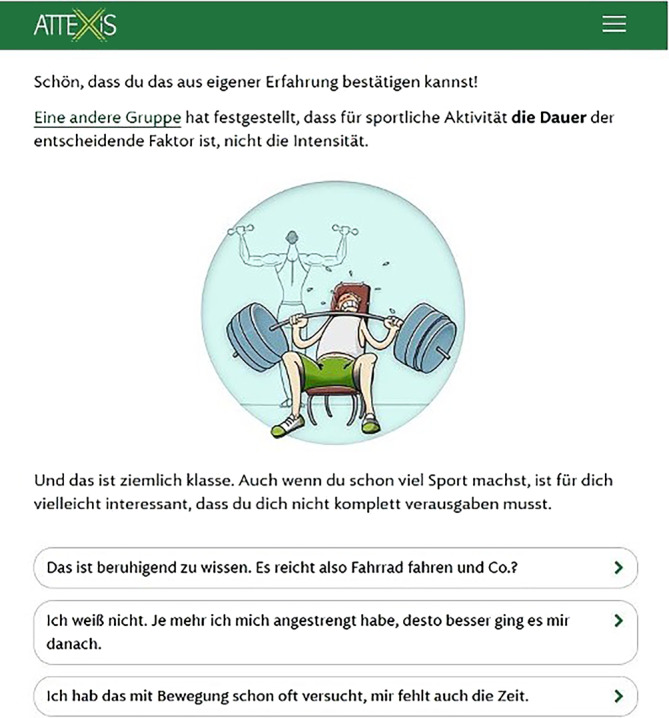
Bildschirmansicht der App attexis mit einem Dialogbeispiel. (Mit freundlicher Genehmigung, © GAIA AG)

Die Wirksamkeit von attexis wurde in einer klinischen Studie mit 337 Erwachsenen mit ADHS untersucht. Nach 3 Monaten wies die Gruppe der Teilnehmenden, die attexis zusätzlich zur üblichen Versorgung erhielt, signifikant geringere ADHS-Symptome auf als die Kontrollgruppe [9]. Die App ist bereits als CE-gekennzeichnetes Medizinprodukt zertifiziert und wurde zur Prüfung beim BfArM eingereicht. Sie wird bei positivem Prüfergebnis voraussichtlich 2025 als verordnungsfähiges Onlineprogramm im Rahmen des DiGA-Verfahrens bereitgestellt (Abb. 1).

### AwareMe-App

Die AwareMe-App wurde innerhalb des BMBF(Bundesministerium für Bildung und Forschung)-geförderten Drittmittelprojekts AwareMe (https://awareme.de/) entwickelt. Die App bietet wissenschaftlich fundierte Psychoedukationsinhalte in mehreren Modulen an und richtet sich primär an erwachsene Patient:innen in der initialen Behandlungsphase der ADHS. Als wichtiger Bestandteil der ADHS-Behandlung hilft die Psychoedukation den Betroffenen, die Erkrankung besser zu verstehen und erste Bewältigungsstrategien zu entwickeln.

#### Kernfunktionen und Nutzungsmodus

**Abb. 2:**
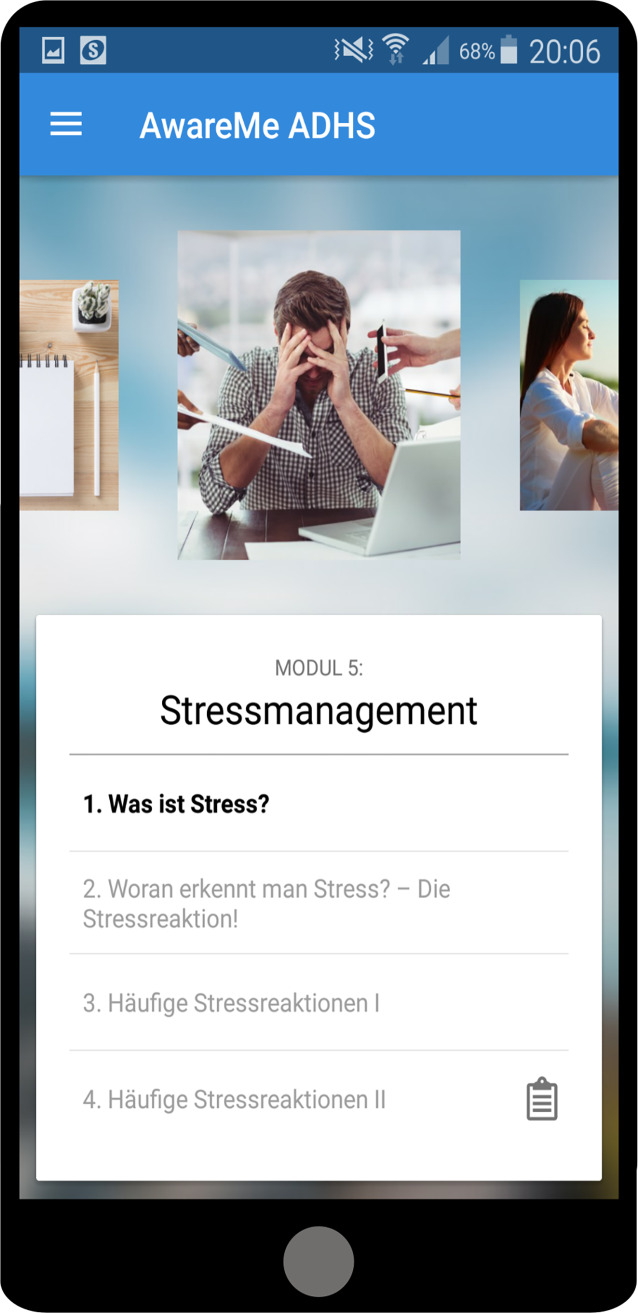
Menüansicht des Moduls zum Thema Stressmanagement der AwareMe-App. (Adaptiert aus [16])

Die App bietet acht Module mit umfassenden Informationen und Symptommanagementstrategien zu Themen wie z. B. persönliche Ressourcen, Selbstorganisation, Stressbewältigung (Abb. 2), Stimmungsregulierung und Impulskontrolle. Jedes Modul enthält zudem Aufgaben zum vertieften Lernen und zur praktischen Anwendung und ein Quiz zur Überprüfung des Lernerfolgs. Das Psychoedukationsmaterial basiert auf einem Handbuch für Psychoedukationsgruppen für Erwachsene mit ADHS [3]. Die App ermöglicht es, die Inhalte flexibel zu bearbeiten.

#### Evidenz- und Reifegrad

Eine Wirksamkeitsprüfung wurde im Rahmen einer randomisiert-kontrollierten Studie durch das Universitätsklinikum Bonn durchgeführt [16]. Insgesamt wurden 60 erwachsene Patient:innen mit ADHS zwei Psychoedukationsgruppen zugeteilt, die entweder durch Psychoedukationsmaterial über die AwareMe-App oder klassische Broschüren in Papierform begleitet wurden [16]. Die Studienergebnisse zeigen, dass die Nutzung der App zu einer stärkeren Verbesserung der ADHS-Symptome führte als die Verwendung traditioneller Psychoedukationsbroschüren. Die App wurde von den Teilnehmenden positiv angenommen, und es traten keine unerwünschten Nebenwirkungen auf. Eine DiGA-Zulassung ist zum aktuellen Zeitpunkt nicht geplant.

### AwareMe-ADHS-Chatbot

Ebenfalls im Rahmen des AwareMe-Projekts wurde ein Chatbot entwickelt, um Erwachsene mit ADHS dabei zu unterstützen, digitale Psychoedukationsinhalte möglichst effektiv selbstständig erarbeiten zu können. Analog zur AwareMe-App vermittelt der Chatbot fundierte Informationen über die Störung, ihre Symptome und Behandlungsmöglichkeiten. Der Chatbot ist gegenwärtig nicht öffentlich verfügbar.

#### Kernfunktionen und Nutzungsmodus

Der Chatbot interagiert mit den Nutzenden in Form eines Dialogs. Die Psychoedukationsinhalte wurden aus der oben beschriebenen AwareMe-App in ein dialogähnliches Format umgeschrieben, um einen möglichst natürlichen Sprachfluss mit dem Chatbot zu ermöglichen. Der Chatbot verwendet in seiner aktuellen Version keine generative künstliche Intelligenz (KI), sondern folgt vorgegebenen Gesprächspfaden. Auf diese Weise werden unerwartete Reaktionen der KI ausgeschlossen, die insbesondere im Bereich der Behandlung psychischer Störungen unerwünscht sind. Überdies bietet der Chatbot den Nutzenden alle Themen der Psychoedukation im Verlauf des Dialogs an und überlässt es dann diesen, sich eingehender mit individuell interessanten Themen zu befassen. Dies soll die Motivation, sich mit den Themen auseinanderzusetzen, steigern und die Therapieadhärenz verbessern.

#### Evidenz- und Reifegrad

In einer randomisiert-kontrollierten Studie wurden 40 erwachsene Patient:innen mit ADHS entweder dem AwareMe-Chatbot oder der in der Vorstudie validierten AwareMe-App [16] zugeteilt und auf Unterschiede in ihrer klinischen Wirksamkeit im Rahmen einer 3‑wöchigen, selbständigen Bearbeitung hin untersucht [15]. Die Ergebnisse zeigen, dass in beiden Gruppen die ADHS-Symptome reduziert wurden, es aber keine signifikanten Unterschiede zwischen den beiden digitalen Formaten gab. Die Ergebnisse deuten an, dass sowohl die künftige Nutzung von Chatbot-basierten als auch herkömmlichen Psychoedukations-Apps, insbesondere in frühen Behandlungsphasen, eine nützliche Unterstützung im Therapieprozess darstellen könnten.

Zu beachten ist allerdings, dass es sich bei der Studie nicht um eine prospektiv geplante und gepowerte Nichtunterlegenheitsstudie handelt, sodass das Fehlen signifikanter Unterschiede zwischen den Gruppen keine abschließende Evidenzaussage im Sinne einer Nichtunterlegenheit des AwareME-Chatbots erlaubt. Folgestudien wären insbesondere zur weiteren Spezifizierung von Subgruppen interessant.

### hiFoon

Die App hiFoon, auch unter dem Vorläufer fuzzymind bekannt (https://www.fuzzymind.de/), soll Erwachsene mit ADHS bei ihrer Emotionsregulation unterstützen. Auch für Menschen mit komorbiden Störungen wie Angst- oder affektive Erkrankungen kann ein ergänzender Nutzen bestehen.

#### Kernfunktionen und Nutzungsmodus

**Abb. 3:**
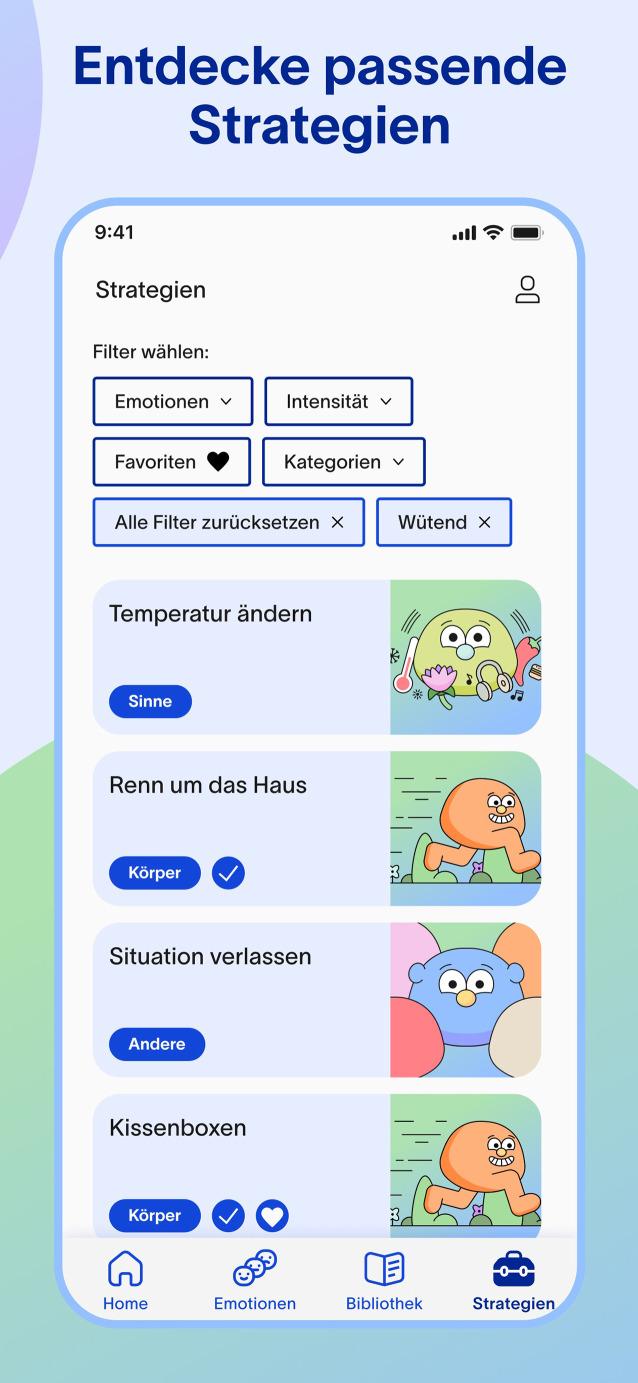
Bildschirmansicht der App hiFoon mit Übersicht verschiedener Strategien zur Emotionsregulation. (Mit freundlicher Genehmigung, © Medigital GmbH)

hiFoon kombiniert verhaltenstherapeutische Ansätze mit Elementen der dialektisch-behavioralen Therapie (DBT; [12]) und der Akzeptanz-und-Commitment-Therapie (ACT; [10]). Der Schwerpunkt von hiFoon liegt auf der Förderung von Emotionskontrolle, Selbstregulation und Achtsamkeit. Die App bietet neben psychoedukativen Inhalten zu ADHS und Emotionsregulation eine Vielzahl an Funktionen wie z. B. ein Emotionstagebuch, Verhaltensanalysen, eine Wissensdatenbank sowie eine Strategie- und Emotionsbibliothek (Abb. 3).

Eine besondere Funktion ist ein physischer Emotionstracker: Nutzende können hierüber ihre Emotionen im Alltag per Tastendruck erfassen. Für die als störend empfundenen Emotionen erhalten die Anwender:innen dann gezielt Vorschläge über evidenzbasierte Bewältigungsstrategien. Die App soll perspektivisch auch in weiteren Sprachen verfügbar sein.

#### Evidenz- und Reifegrad

Erste Datenauswertungen aus einer Anwender:innenbefragung an Personen der Allgemeinbevölkerung vor und nach 6 Wochen App-Nutzungsphase deuten auf Verbesserungen im Bereich der Emotionsregulation und Lebensqualität hin. Weitere Befragungen sowie eine randomisiert-kontrollierte Studie sind in Planung (mündliche Referenz). Eine Listung im DiGA-Verzeichnis wird angestrebt.

### NeuroNation MED

Die App NeuroNation MED ist ein Medizinprodukt und richtet sich an Patient:innen mit verschiedenen psychischen und/oder neurologischen Störungen (https://neuronation-med.de), einschließlich ADHS. Die Anwendung soll Patient:innen dabei unterstützen, kognitive Beeinträchtigungen gezielt durch Trainingsprogramme zu lindern.

#### Kernfunktionen und Nutzungsmodus

**Abb. 4:**
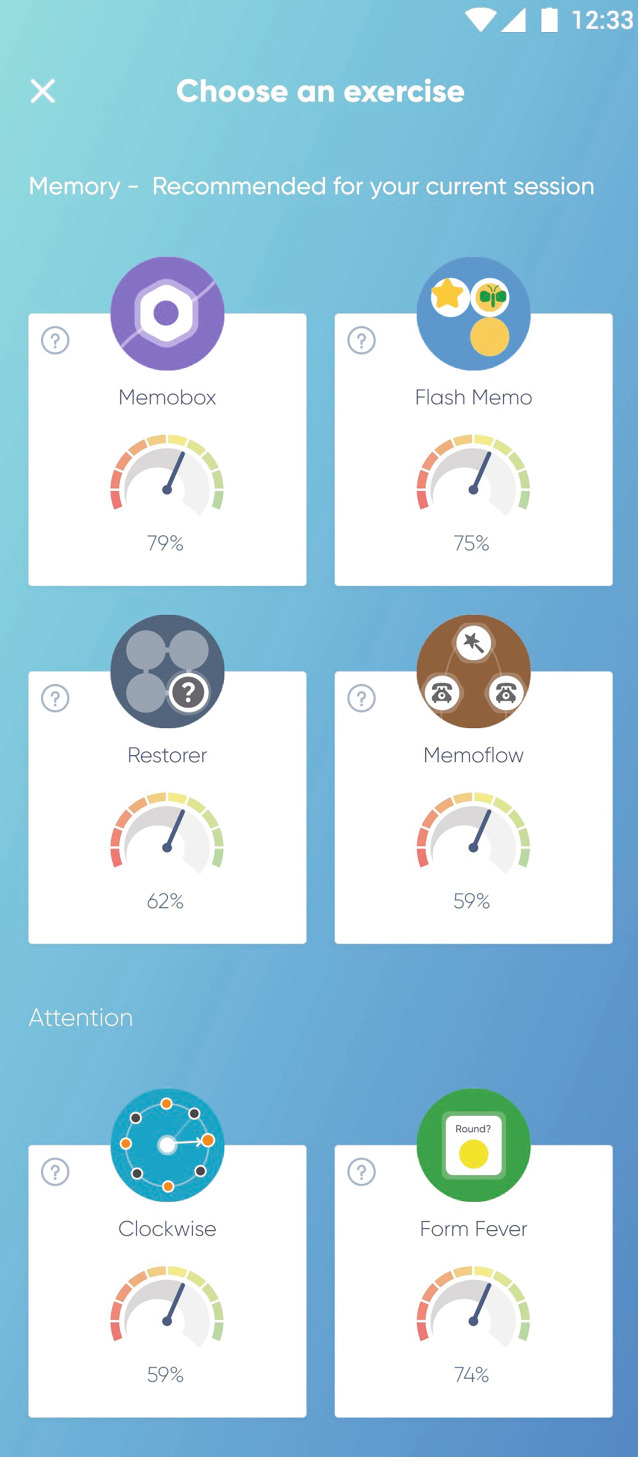
Bildschirmansicht mit Übersicht verschiedener Übungen der App NeuroNation MED. (Mit freundlicher Genehmigung, © NeuroNation MED)

NeuroNation MED bietet individuell anpassbare Übungen zur Förderung von Gedächtnis, Aufmerksamkeit, Exekutivfunktionen und anderen kognitiven Bereichen (siehe z. B. Abb. 4). Der Therapieansatz basiert auf der Evaluation eines persönlichen kognitiven Profils und der algorithmischen Zusammenstellung kognitiver Trainingssitzungen aus einem Set multimodaler Übungen. Zudem bieten Gesundheitsinformationen, Stressmanagement- und Entspannungstechniken Hilfestellung bei der Emotionsregulation. Die Übungen basieren auf wissenschaftlichen Erkenntnissen und sind so gestaltet, dass sie die Patient:innen schrittweise herausfordern. Darüber hinaus ermöglicht die Anwendung eine genaue Auswertung der Fortschritte, die von den Behandler:innen genutzt werden können, um den Therapieverlauf zu überwachen.

#### Evidenz- und Reifegrad

Die Anwendung ist CE-gekennzeichnet und erfüllt die regulatorischen Anforderungen gemäß Medizinprodukterecht-Durchführungsgesetz (MPDG). Aktuell ist die App für die leichte kognitive Störung (F06.7) als DiGA von den Krankenkassen voll erstattungsfähig [2]. Die Erstattung für ADHS wird angestrebt. Die DiGA NeuroNation MED ist in den Sprachen Deutsch und Englisch verfügbar.

### ORIKO

ORIKO ist eine digitale Intervention, die speziell für Erwachsene mit ADHS entwickelt wurde und eine Unterstützung im Alltag der Betroffenen bietet (https://www.oriko-adhs.de).

#### Kernfunktionen und Nutzungsmodus

**Abb. 5:**
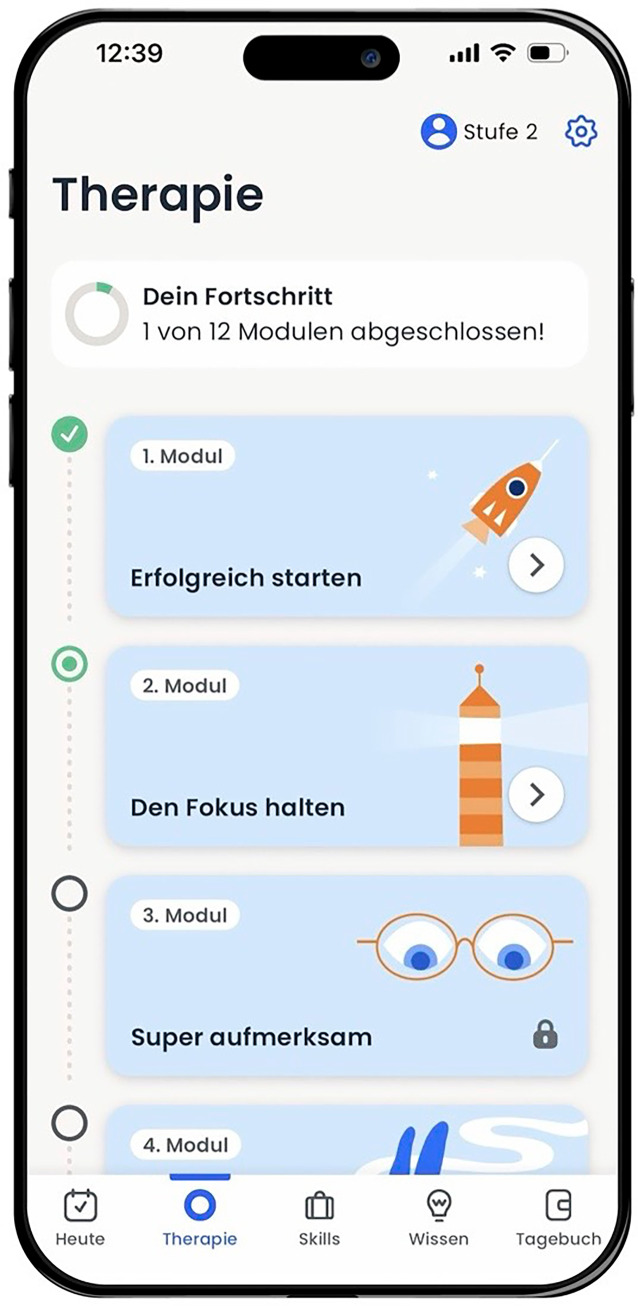
Bildschirmansicht des Menüs der App ORIKO. (Mit freundlicher Genehmigung, © MiNDNET e‑Health AG)

Die App bietet ein 12-wöchiges geführtes Therapieprogramm (Abb. 5), das Grundlagenwissen über ADHS vermittelt und alltagsrelevante Fertigkeiten fördern soll. Die aufeinander aufbauenden Therapiemodule beinhalten verhaltenstherapeutische Übungen und Skills-Trainings und widmen sich Themen wie z. B. Aufmerksamkeitslenkung, effektive Zeitsteuerung, soziale Kompetenzen oder Selbstfürsorge. Die App wird dabei durch ein begleitendes Arbeitsbuch unterstützt. Weitere Funktionen umfassen Medikamentenerinnerungen, einen digitalen ADHS-Test und ein Tagebuch zur Dokumentation von Gedanken.

#### Evidenz- und Reifegrad

Eine randomisiert-kontrollierte Pilotstudie lieferte erste Evidenz für eine Reduktion von ADHS-Symptomen und eine signifikante Verbesserung der ADHS-bezogenen Lebensqualität bei mittlerer Effektstärke [14]. ORIKO wurde am 09.07.2025 nach positiver Prüfung durch das BfArM in das DiGA-Verzeichnis aufgenommen (https://diga.bfarm.de/de/verzeichnis/02568). Diese DiGA kann nun für den Indikationsbereich ADHS auf Rezept verordnet werden [18].

## Diskussion

In dieser Literaturübersicht wurden die aktuell in Deutschland verfügbaren sowie sich in der Entwicklung befindlichen digitalen Interventionen für Erwachsene mit ADHS vorgestellt. Während einige der digitalen Interventionen eine zeitnahe Listung im DiGA-Verzeichnis anstreben (attexis, hiFoon, NeuroNation MED), ist bisher nur eine der vorgestellten Interventionen als verschreibbare DiGA für den Indikationsbereich ADHS vorläufig aufgenommen (ORIKO). Die für eine (dauerhafte) DiGA-Zulassung notwendigen Wirksamkeitsnachweise werden für einzelne der vorgestellten digitalen Interventionen aktuell im Rahmen laufender oder geplanter klinischer Studien geprüft.

Die meisten Apps basieren auf evidenzbasierten Ansätzen und unterstützen Menschen mit ADHS bei der eigenständigen Symptomregulation durch therapeutische Methoden und Selbstmanagement. Sie eignen sich sowohl zur alleinigen Nutzung als auch als Teil einer multimodalen Behandlung, wobei Studien meist die eigenständige Anwendung untersuchen. Inhaltliche Schwerpunkte liegen in der Vermittlung psychoedukativer Inhalte und verhaltenstherapeutischer Methoden entweder zu ADHS im Allgemeinen (z. B. bei attexis, ORIKO, AwareMe-App/Chatbot) oder zu bestimmten Symptombereichen (z. B. Emotionsregulationsfähigkeiten bei hiFoon u. a. mit einem individuellen Emotionstracker oder kognitive Beeinträchtigungen bei NeuroNation MED). Unterschiede werden vor allem hinsichtlich der Nutzungsmodi deutlich: Während einige der Anwendungen Inhalte dialogbasierend vermitteln (z. B. attexis, AwareMe-Chatbot), setzen andere auf einem modularen Aufbau (z. B. hiFoon, ORIKO), welcher aber zum Teil individualisierbar ist oder durch interaktive Funktionen ergänzt wird (z. B. hiFoon).

Insgesamt bieten die vorgestellten digitalen Interventionen vielversprechende Funktionen und können zur Unterstützung von Menschen mit ADHS beitragen. Weitere Forschung ist allerdings erforderlich, um ihre Wirksamkeit in der ADHS-Behandlung und ihren langfristigen Einfluss auf die Lebensqualität der Nutzenden zu belegen.

### Innovationspotenziale und Ausblick

Die Entwicklung digitaler Interventionen zur Behandlung von ADHS befindet sich noch in einem frühen Stadium und erfordert weitere konzeptionelle und empirische Fortschritte. Ein Vorteil digitaler Interventionen ist beispielsweise die Möglichkeit der niederschwelligen Umsetzung multilingualer Interventionen. Da die meisten der hier vorgestellten digitalen Interventionen bisher nur auf Deutsch verfügbar sind, könnte eine Übersetzung den potenziellen Nutzer:innenkreis deutlich erweitern.

Auch könnten die digitalen Interventionen um Mobile-Sensing-Funktionen erweitert werden. Typische Sensordaten, die hierfür genutzt werden könnten, umfassen u. a. Standortinformationen (z. B. das Betreten eines bestimmten Ortes), Bewegungs- und Aktivitätsmuster (z. B. zurückgelegte Schrittzahl) sowie verhaltensbezogene Daten (z. B. Social-Media-Nutzung). Durch den Einbezug solcher Datenquellen könnten kontextangereicherte und ereignisgesteuerte Problem- und Verhaltensanalysen sowie individualisierte therapeutische Interventionen („just-in-time adaptive interventions“) durchgeführt werden. Für die Behandlung der ADHS, bei der die Etablierung funktionaler Automatismen wichtig ist, könnte die Nutzung von Mobile Sensing besonders gewinnbringend sein.

## Fazit für die Praxis

- In der Versorgung von Erwachsenen mit Aufmerksamkeitsdefizit‑/Hyperaktivitätsstörung (ADHS) kommt der Psychoedukation sowie der Etablierung funktionaler Alltagsroutinen eine große Bedeutung zu.
- Digitale Interventionen, die grundsätzlich unabhängig von Zeit und Ort genutzt werden können, bieten hier das Potenzial, entsprechende Psychoedukation zu vermitteln sowie Verhaltensänderungen kleinschrittig im Alltag anzuleiten.
- Aktuell lässt sich lediglich die DiGA ORIKO auf Rezept verordnen. Weitere Anwendungen streben ebenfalls eine zeitnahe Listung im DiGA-Verzeichnis an. Eine evidenzbasierte Empfehlung ist erst mit Erreichen der dauerhaften Listung möglich, da diese einen nachgewiesenen positiven Versorgungseffekt voraussetzt.
- Innovationspotenziale liegen in der Übersetzung bestehender Anwendungen sowie der Integration von Mobile-Sensing-Funktionen.

## Supplementary Information

Infobox: Wichtige Begriffe in der digitalen Gesundheitsversorgung im Bereich Psychiatrie und Psychotherapie

Tabelle 1: Digitale Interventionen für Erwachsene mit ADHS.
