# Supplementary material for: Therapy and healthcare applications in the treatment of adult attention deficit hyperactivity disorder: a review
Source: Nervenarzt. 2025 Aug 4;96(5):451–7. [Article in German] doi: 10.1007/s00115-025-01855-1 (PMC12411583; doi:10.1007/s00115-025-01855-1)
Supplement: Supplementary file 2 — Tabelle 1: Digitale Interventionen für Erwachsene mit ADHS. [file 115_2025_1855_MOESM2_ESM.docx]

Tab e1: Digitale Interventionen für Erwachsene mit ADHS.

| **Name** | **Unternehmen/**  **kooperierende Forschungsinstiutionen** | **Therapeutischer Fokus** | **Umfang/Bearbeitungsdauer** | **Plattform** | **Evidenzbasis** | **DiGA-Zulassung / Verfügbarkeit** |
| --- | --- | --- | --- | --- | --- | --- |
| attexis | Gaia (Hersteller),  Medice*^2^*  (Vertriebspartner), UKS, ZIP Kiel | Symptom-bewältigung | Virtueller Dialog (empfohlene Nutzungsdauer: mind. 90 Tage) [8] | Web-Anwendung | Eine randomisiert-kontrollierte klinische Studie (N = 337; [13]*^1^*) | Geplante DiGA-Listung |
| AwareMe ADHS App | UKB, OFFIS, Ascora | Psycho-  edukation | 8 Module (Dauer: individuell) | Smartphone-App für Android-Geräte | Eine randomisiert-kontrollierte Studie (N = 60; [16]) | Nein  Verfügbar über: Google Play Store |
| AwareMe ADHS Chatbot | UKB, OFFIS, Ascora | Psycho-  edukation | Inhalte aus 8 Modulen (Dauer: individuell) | Web-Anwendung | Eine randomisiert-kontrollierte Studie (N = 40; [15]) | Nein  Derzeit nicht frei verfügbar |
| hiFoon | Medigital*^2^* / UKB | Emotions-regulations-  fähigkeiten | Auswahl an Modulen (Dauer: 12 Wochen) | Smartphone-App mit smartem Hardware Element (EMA) | Pilotierung: Anwender:innenbefragung & App-Testung u. geplante randomisiert-kontrollierte klinische Studie (N = 212) | Geplante DiGA-Listung |
| NeuroNation MED | Synaptikon GmbH / CB, FUB, UKJ, UKK, UKB, TUD, MSH, UL, LMU, CHNP, AK | Kognitive Fähigkeiten | Empfohlene Nutzung: 30 Minuten an 3 Tagen pro Woche (Dauer: 12 Wochen) | Smartphone-App | Laufende klinische Studie (N = 78; [5]*^1^*) | Geplante DiGA-Listung, derzeit vorläufige Zulassung für die Diagnose F06.7 (leichte kognitive Störung)  Verfügbar über: Apple App Store, Google Play Store |
| ORIKO | Takeda & MiNDNET / AKH, UKHE, CB, UKF, MHH, UKB | Symptom-bewältigung | 12 Module (Dauer: 12 Wochen) | Smartphone-App mit begleitendem Arbeitsbuch | Eine randomisiert-kontrollierte klinische Studie (N = 307; [14]*^1^*) | DiGA-Listung seit 09.07.2025 |

Anmerkungen: UKS = Universitätsklinikum Saarland, ZIP Kiel = Zentrum für integrative Psychiatrie, Kiel, UKB = Universitätsklinikum Bonn, CB = Charité-Berlin, FUB = Freie Universität Berlin, UKJ = Universitätsklinikum Jena, UKK = Universitätsklinikum Köln, TUD = Technische Universität Dortmund, MSH = Medical School Hamburg, UL = Universität Leipzig, LMU = Ludwig-Maximilians-Universität München, CHNP = Centre Hospitalier Neuro-Psychiatrique au Luxembourg, AK = Asklepios Kliniken, AKH = Asklepios Klinikum Harburg, UKHE = Universitätsklinikum Hamburg-Eppendorf, UKF = Universitätsklinikum Frankfurt, MHH = Medizinische Hochschule Hannover)

^1^ Daten derzeit nicht veröffentlicht.

^2^ Medigital GmbH (Hersteller) = Tochterfirma der MEDICE Arzneimittel Pütter GmbH & Co. KG
